# Patients' Experience of Using Virtual Consultations in Their Care During Their Inpatient Stay on Acute Mental Health Admission

**DOI:** 10.1192/bjo.2024.483

**Published:** 2024-08-01

**Authors:** Ahmed Elshafei, Elizabeth Junaid, Umoh Ekwere, Fatima Maheen

**Affiliations:** KMPT NHS Trust, Canteurbury, United Kingdom

## Abstract

**Aims:**

To obtain the views of patients regarding their experience of meetings where virtual media (video conferencing) has been used during their inpatient stay in the acute female admission ward.

**Methods:**

Data was collected via a questionnaire. Service users who met the inclusion criteria were past and current inpatients in the acute female psychiatric ward during the last six months. The sample of the service users included in the project was selected from all applicable cases via convenience sampling – those on the ward who consented and were able to engage, as well as past inpatients whom we contacted via telephone after their discharge who met these same criteria.

Verbal consent was obtained from all the patients who agreed to participate. Data was collected and analysed using Microsoft Excel.

**Results:**

13 patients in total completed the facilitated questionnaire which used 11 questions rated by Likert Scale as well as an open space area for further comments. Age ranges varied among participants with 39% age range 18–30, 38% aged 31–50 and 23% aged 51–65. 61% were of white British descent. Majority (38%) were admitted for schizophrenia, schizotypal and delusional disorders, 31% for disorders of adult personality, 23% for mood (affective) disorder and 8% for anxiety, dissociative, stress related, somatoform and nonpsychotic mental disorders.

Most patients rated the use of virtual consultations positively, with over ¾ of patients answering strongly agree or agree (positive response) to most questions. This included feeling able to express themselves effectively as in an in-person consultation, feeling that they received adequate care, feeling that the audio-visual quality was satisfactory and that their privacy was respected. One suggestion for improvement from the patients was to clarify the number of people in the room and how many students are present during the consultation.

**Conclusion:**

Virtual consultations were overall well received among the patients interviewed. Interventions that facilitate timeliness and privacy in consultations as well as training for staff in verbal and nonverbal communication skills for virtual consultations would be beneficial. Further surveys in groups underrepresented in the survey such as men, older people, ethnic minority groups, people with visual or hearing impairment and other mental disorders not present in the sample would help to give further insight into how virtual consultations are received and barriers by different groups.

